# Prospective studies of allogeneic hema topoietic stem cell transplantation for adult T-cell leukemia-lymphoma

**DOI:** 10.1186/1742-4690-12-S1-O16

**Published:** 2015-08-28

**Authors:** Ilseung Cho, Atae Utsunomiya, Ryuji Tanosaki, Tetsuya Eto, Takahiro Fukuda, Youko Suehiro, Mari Kannagi, Jun Okamura, Naokuni Uike

**Affiliations:** 1 Department of Hematology, National Kyushu Cancer Centre, Fukuoka, Japan; 2 Department of Hematology, Imamura Bun-in Hospital, Kagoshima, Japan; 3 Clinical Laboratory Division, National Cancer Centre Hospital, Tokyo, Japan; 4 Department of Hematology, Hamanomachi Hospital, Fukuoka, Japan; 5 Division of Hematopoietic Stem Cell Transplantation, National Cancer Center Hospital, Tokyo, Japan; 6 Department of Immunotherapeutics, Tokyo Medical and Dental University, Tokyo, Japan; 7 Institute for Clinical Research, National Kyushu Cancer Centre, Fukuoka, Japan

## 

Adult T-cell leukemia-lymphoma (ATL) is divided into two types for decision of therapeutic strategy; aggressive variant includes acute, lymphoma and chronic type with poor prognostic factor, and indolent variant includes smoldering and chronic type without any factors. Prognosis of aggressive ATL by chemotherapy alone is very poor. We have reported the possibility of improvement of prognosis in aggressive ATL by allogeneic hematopoietic stem cell transplantation (allo-HSCT) for the first time in 2001, however transplant-related mortality (TRM) was very high. In order to reduce TRM and improve outcomes of aggressive ATL, we conducted prospective clinical trials of allo-HSCT using the reduced intensity conditioning (RIC). The first clinical trial (NST-1) was performed for evaluating allo-HSCT with RIC using HLA matched sibling donor in the patients of aggressive ATL aged over 50, and subsequently NST-2 trial was applied for the same eligibility. Conditioning regimen in NST-1 included busulfan, fludarabine and antithymocyte globulin (ATG), but omitted ATG in NST-2 because of early relapse. In all 29 patients registered in NST-1 and -2, 3-year and 5-year overall survival rates (OS) were 36% and 34% respectively. Eight patients were died of TRM. Ten of the 29 patients obtained long-term survival with good performance status. Subsequently, NST-3 trial was conducted with similar eligibility with NST-2 to confirm the efficacy of the therapeutic strategy of RIC. Twenty patients were transplanted and 2-year OS was 53%. The availability of sibling donors is becoming difficult mainly due to the aging of not only patients but also donors. Therefore, NST-4 trial was performed with similar eligibility using unrelated bone marrow (UBM) as alternative stem cell source. Fifteen patients were transplanted, we have confirmed the feasibility of UBM and 2-year OS was 67%. For the further improvement of allo-HSCT for aggressive ATL, the study using cord blood (NST-5) is on the way.

